# Maternal sevoflurane exposure affects differentiation of hippocampal neural stem cells by regulating miR-410-3p and ATN1

**DOI:** 10.1186/s13287-020-01936-9

**Published:** 2020-09-29

**Authors:** Yi Zhang, Ziyi Wu, Xingyue Li, Yuxiao Wan, Yinong Zhang, Ping Zhao

**Affiliations:** Department of Anesthesiology, Shengjing Hospital of China Medical University, Shenyang, China

**Keywords:** Sevoflurane, Mid-trimester, Neural stem cells, Differentiation, Neurotoxicity, microRNA

## Abstract

**Background:**

Currently, numerous animal studies have shown that exposure to commonly used general anesthetics during pregnancy may cause neurocognitive impairment in the offspring. Reportedly, exposure to sevoflurane during mid-trimester of pregnancy can inhibit proliferation of neural stem cells (NSCs) and lead to early apoptosis. Whether exposure to sevoflurane during pregnancy affects the differentiation of NSCs remains unclear.

**Methods:**

In the present study, pregnant rats were exposed to 3% sevoflurane once for 2 h on gestational day 14 (G14) or 3 times for 2 h on G13, G14, and G15. Next, the differentiation of NSCs was measured using neuron marker β-tubulin III and astrocyte marker glial fibrillary acidic protein (GFAP) in fetal brain tissues 24 h and 72 h after anesthesia and in hippocampus on postnatal day 28. Primary cultured rat NSCs were exposed to 4.1% sevoflurane to explore the mechanism.

**Results:**

The results showed that during mid-trimester, multiple exposures to sevoflurane can cause premature differentiation of NSCs in developing brains of offspring and lead to long-term neuron reduction and astrocyte proliferation in hippocampus. The data from the present study indicated that repeated exposure to sevoflurane downregulated atrophin-1 (ATN1) expression and caused early differentiation of NSCs. Overexpression of ATN1 via lentivirus transfection attenuated the influence of sevoflurane. Using dual luciferase assay, ATN1 was found to be a target gene of microRNA-410-3p (miR-410-3p). MiR-410-3p suppression via lentivirus transfection recovered the ATN1 expression and differentiation of NSCs.

**Conclusions:**

The results from the present study demonstrated that repeated exposure to sevoflurane leads to early differentiation of NSCs and long-term effects via the miR-410-3p/ATN1 pathway.

## Background

Numerous animal studies have shown that exposure to anesthesia drugs can lead to long-term neurocognitive impairment in the developing brain. Most research has focused on the newborn period [1–3]. Because the surgical technologies are constantly advancing, the number of non-obstetric surgeries is continually increasing. Mid-trimester is considered a relatively safe period to perform surgery during pregnancy. However, the mid-trimester is a critical period for fetal brains because neural stem cells (NSCs) undergo high proliferation and differentiation [4, 5]. In our previous studies [6–8], exposure to sevoflurane in the mid-trimester of pregnancy suppressed NSC proliferation and led to early apoptosis. However, whether sevoflurane affects NSC differentiation remains unclear.

In the present study, the results showed that after sevoflurane exposure, the atrophin-1 (ATN1) level was significantly decreased. ATN1 is highly expressed in brain tissues and mutations in the ATN1 gene can cause a rare neurodegenerative disease, dentatorubral-pallidoluysian atrophy (DRPLA) [9]. In most of the previous ATN1 expression studies [10, 11], the focus was on DRPLA. Recently, ATN1 was reported to play an important role in NSC maintenance [12], which is in agreement with our results. Thus, in the present study, whether sevoflurane could affect NSC differentiation by changing the expression of ATN1 was investigated.

In numerous studies, microRNAs (miRNAs) have been reported to play significant roles in neural development [13, 14]. Several miRNAs were confirmed to participate in NSC apoptosis and differentiation [15, 16]. Abnormal miRNA expression after exposure to anesthesia drugs could cause significant neurocognitive impairments in developing brains [17, 18]. In the present study, several databases were used to determine if ATN1 is a direct target of miRNA-410-3p (miR-410-3p), which was confirmed using dual-luciferase reporter assay. However, whether miR-410-3p plays a role in NSC differentiation after exposure to sevoflurane remains unknown.

To investigate whether sevoflurane affects NSC differentiation during the early stage of brain development, rat models in mid-trimester of pregnancy and primary cultured NSCs were repeatedly exposed to sevoflurane. We hypothesized that repeated sevoflurane exposure would lead to early NSC differentiation and long-term neuron reduction by regulating the miR-410-3p and ATN1 expression.

## Methods

### Animals

Adult Sprague-Dawley rats were housed in a room with constant temperature of 24 ± 1 °C under a 12-h light/12-h dark cycle, with free access to water and food. All experimental procedures were approved by the Institutional Animal Care and Use Committee of Shengjing Hospital, China Medical University (No.2016PS028K), and were conducted following the National Institute of Health Guideline for the Care and Use of Laboratory Animals.

### Cell culture and differentiation

Rat NSCs were isolated from the hippocampus of fetal Sprague-Dawley rats on G14 or G15. The isolated cells were plated in culture flasks at a density of 1 × 10^5^/mL and maintained in an atmosphere of 5% CO_2_ and 95% air. The cells were cultured in serum-free medium of Dulbecco’s modified Eagle’s medium (DMEM)/F12, HEPES (11330032; Gibco, USA) supplemented with 2% B27 without vitamin A (12587010; Gibco), 20 ng/mL basic fibroblast growth factor (rat bFGF, 3339-FB-025; R&D, USA), 20 ng/mL epidermal growth factor (rat EGF, 3214-EG-100; R&D), and 1% penicillin-streptomycin (1512022; Gibco). Half of the culture medium was replaced every other day, and the cells were digested with Accutase (A6964; Sigma-Aldrich, USA). All experiments were performed on cells from passages 2–4 to reduce experimental deviations. To induce NSC differentiation, rat NSCs were digested into single cells and plated onto plates pre-coated with 0.25% poly-D-lysine (P6409; Sigma-Aldrich) using differentiation medium of DMEM/F12 supplemented with 2% B27 without vitamin A, 1% fetal bovine serum (FBS, 10100139; Gibco), and 1% penicillin-streptomycin.

HEK293T cells were purchased from Cell Bank of Chinese Academy of Sciences (Shanghai, China) and cultured in high-glucose DMEM (11965084; Gibco) supplemented with 10% FBS (10100139; Gibco) and antibiotics (1512022; Gibco). Cells were incubated in an atmosphere at 37 °C in 5% CO_2_ and the culture medium replaced every other day.

### Sevoflurane exposure

Two or three adult female Sprague-Dawley rats were caged with one male rat to allow free mating. If sperm or vaginal emboli were detected on the second day, the female rat was marked as G0. For the in vivo experiments, pregnant rats were placed into a 30% oxygen plastic chamber and exposed to 3% sevoflurane (1.5 MAC) for 2 h on G14 (SEV × 1 group; *n* = 9/group) or on G13, G14, and G15 (SEV × 3 group; *n* = 9/group). CON group (*n* = 9/group) consisted of rats placed in the same conditions without exposure to sevoflurane. The fetal brain tissues were obtained after cesarean section at 24 h, 72 h, and postnatal day 28. For the in vitro experiments, hippocampal NSCs were obtained as described above. The NSCs were inoculated into 24-well plates and randomly allocated into 8 groups: CON group, 4.1% SEV × 1 group (SEV × 1), 4.1% SEV × 3 group (SEV × 3), negative lentivirus group (NC), ATN1-overexpression lentivirus group (LV-ATN1), 4.1% SEV × 3 plus ATN1-overexpression lentivirus group (LV-ATN1 + SEV × 3), miR-410-3p-suppression lentivirus group (LV-410), and 4.1% SEV × 3 plus miR-410-3p-suppression lentivirus group (LV-410 + SEV × 3). The plates were placed into an incubator containing a mixture of 5% CO_2_ and 95% air, and a gas monitor (Drager, Germany) was used to detect sevoflurane concentration. The SEV × 1 group was exposed to 4.1% sevoflurane for 2 h, and all SEV × 3 groups were exposed to 4.1% sevoflurane for 2 h on 3 consecutive days. All groups that were not exposed to sevoflurane were kept under the same conditions of a mixture of 5% CO_2_ and 95% air for the same amount of time.

### Western blot

The brain tissues and collected cells were stored at − 80 °C before use and homogenized to determine protein expression using the western blotting protocol described in our previous study [19]. The protein concentration was measured using the BCA Protein Assay Kit (P0010; Beyotime, China). Electrophoresis was performed using 10% or 12.5% SDS-polyacrylamide gels, and then proteins were electrotransferred to polyvinylidene fluoride membranes (IPVH0010; Millipore, Germany). The membranes were blocked with BSA or 5% non-fat milk and incubated with primary antibodies β-tubulin III (1:1000, T2200; Sigma-Aldrich), GFAP (1:10,000, ab53554; Abcam, UK), nestin (1:1000, ab6142; Abcam), ATN1 (1:500, orb213859; Biorbyt, UK), and glyceraldehyde-3-phosphate dehydrogenase (GAPDH) (1:1000, 60004-1-Ig; Proteintech, USA) overnight at 4 °C. The membranes were incubated with second antibodies the next day for 2 h at room temperature, and then photographed using a GE Amersham Imager 600. Number of tissues was 5 per group and number of cells was 3 per group in western blot experiments. Images were analyzed using Image-Pro Plus 6.0 software.

### RT-qPCR

The mRNA in each group was extracted using RNAiso Plus (9108; TaKaRa, China) and miRNA was extracted using RNAiso for small RNAs (9753A; TaKaRa, China). The primers were designed and synthesized by Sangon Biotech (China; Table 1). Reverse transcription and reaction conditions were performed according to the instructions in the PrimeScript™ RT reagent Kit with gDNA Eraser (RR047A; TaKaRa) and Mir-X miRNA First-Strand Synthesis Kit (638,315; Clontech, USA) using SYBR green (RR420A; TaKaRa, China). Quantitative analysis of gene expression was calculated using 2^−ΔΔCt^ method with GAPDH (B661204; Sangon biotech, China) and U6 as internal references.

**Table 1:** Primer sequences for RT-qPCR

Gene	Sequence
ATN1	Forward: GTCTTCGTCTCAAGCCGCCTATTC Reverse: AGGAGGTGGTGATTGGAGGAACTG
miR-410-3p	cgcgAATATAACACAGATGGCCTGT

### Immunofluorescence staining

Brains were immersed in precooled 4% paraformaldehyde (PFA) for 24 h followed by dehydration in an ethanol gradient, and then paraffin embedded. Next, the tissues were sliced into sections approximately 3.0-μm-thick. The sections were deparaffinized and heated in citrate buffer for 7.5 min at 121 °C. The cells were plated onto glass coverslips pre-coated with poly-D-lysine, and then fixed in 4% PFA for 30 min at room temperature. To reduce background staining and permeabilize membranes, 10% FBS and 0.5% Triton X-100 were added to the sections and coverslips for 40 min at room temperature, and then incubated with primary antibodies overnight at 4 °C. The next day, the sections were incubated with secondary antibodies for 2 h and DAPI for 5 min at room temperature. The primary antibodies were the following: nestin (1:250, ab92391; Abcam; 1:300, 4760; Cell Signaling Technology, USA), sex-determining region Y box 2 (SOX2) (1:500, abs131219-50ug; absin), β-tubulin III (1:250, T2200; Sigma-Aldrich), GFAP (1:500, ab53554; Abcam), and ATN1 (1:500 HPA031619; Sigma-Aldrich). The nuclei were stained with DAPI. Sections and coverslips were imaged with Nikon C1 microscope by an investigator who was blinded to the experimental interventions. The β-tubulin III and GFAP immunoreactivity was quantified by the ratio of the number of β-tubulin III or GFAP-positive cells to the total number of cells. Number of rats per group was 5 (*n* = 5) and number of cell coverslips per group was 3 (*n* = 3).

### Lentivirus transfection

ATN1 overexpression lentivirus and miR-410-3p suppression lentivirus were purchased from GeneChem Corporation, China. NSCs in 96-well plates were transfected to detect the optimum multiplicity of infection (MOI) and experiments performed in 24-well plates. Medium containing lentivirus was replaced 24 h post-transfection with fresh medium. Transfection efficiency was confirmed using RT-qPCR.

### Dual-luciferase reporter assay

Dual-luciferase reporter assay was performed in 293 T cells using the Dual-luciferase Reporter System (Promega, China). All pmirGLO vectors were purchased from GenePharma, China. The pmirGLO-ATN1-WT or pmirGLO-ATN1-MUT vector was transfected with miR-410-3p mimics or mimics negative control (mimics-NC) into 293 T cells using Lipo2000 (Invitrogen, USA). Luciferase activity was detected at 24 h after transfection. The experiments were performed three times independently.

### Statistical analysis

All data were analyzed using GraphPad Prism 7.0 software or SPSS 17.0 for Windows. Results were presented as means ± standard deviation (SD). Student’s *t* test and one-way analysis of variance (ANOVA) followed by Tukey’s post hoc multiple comparison test was used for data analyses. A *p* value < 0.05 was considered statistically significant.

## Results

### Confirmation and differentiation of NSCs

**Fig. 1:**
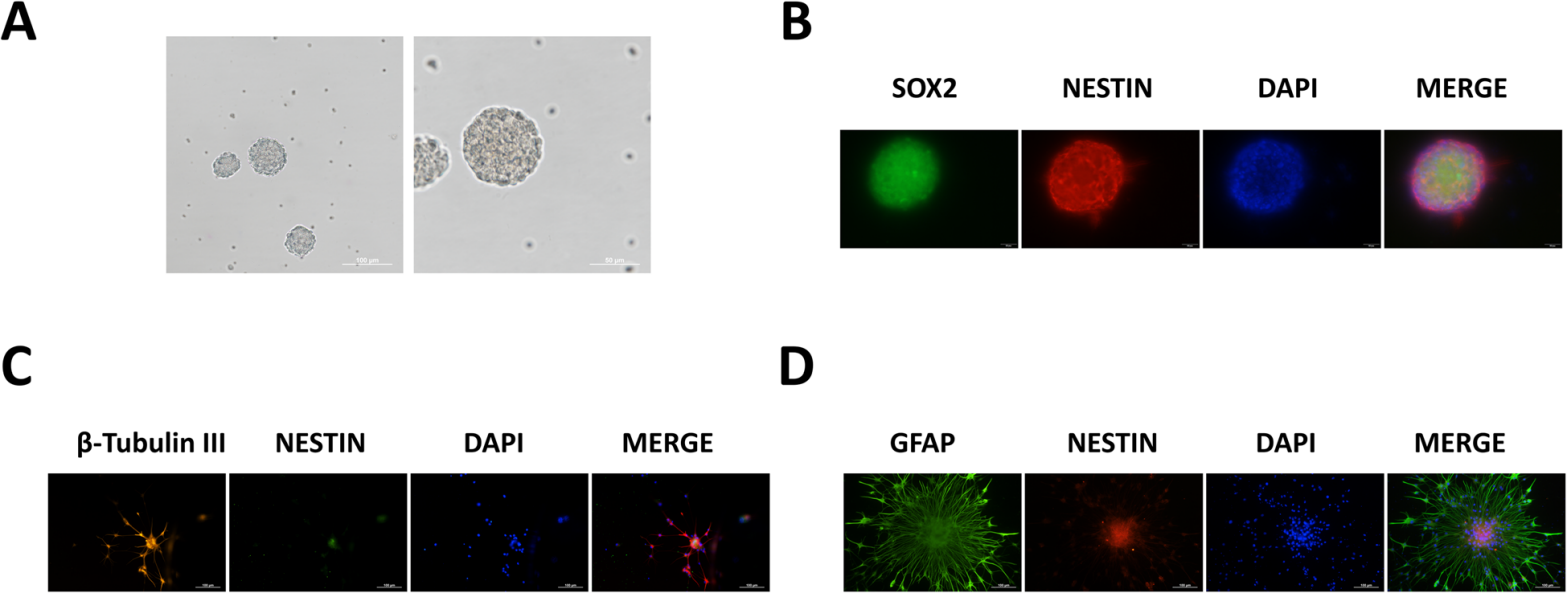
Confirmation and differentiation of neural stem cells (NSCs). **a** Primary cultured hippocampal NSCs observed under microscope. Scale bar = 100 μm, 50 μm. **b** Immunofluorescence images of NSC marker nestin (red) and Sex-determining region Y box 2 (SOX2) (green). Scale bar = 20 μm. **c** Immunofluorescence images of β-tubulin III (red)and nestin (green). Scale bar = 100 μm. **d** Immunofluorescence images of glial fibrillary acidic protein (GFAP) (green) and nestin (red). Scale bar = 100 μm

To investigate the mechanism in vitro, primary cultured hippocampal NSCs were used. The NSCs were isolated from fetal hippocampi of Sprague-Dawley rats on embryonic (E) day 14 to E15. Observation under microscope showed that NSCs grew in a typical round shape (Fig. 1a), which was confirmed using immunofluorescence with the NSC marker nestin and Sex-determining region Y box 2 (SOX2)(Fig. 1b). For NSC differentiation, NSCs were induced to differentiate for 120 h which was confirmed using immunofluorescence with neuron marker β-tubulin III (Fig. 1c) and astrocyte marker glial fibrillary acidic protein (GFAP; Fig. 1d).

### Repeated exposure to sevoflurane led to early differentiation of hippocampal NSCs

**Fig. 2:**
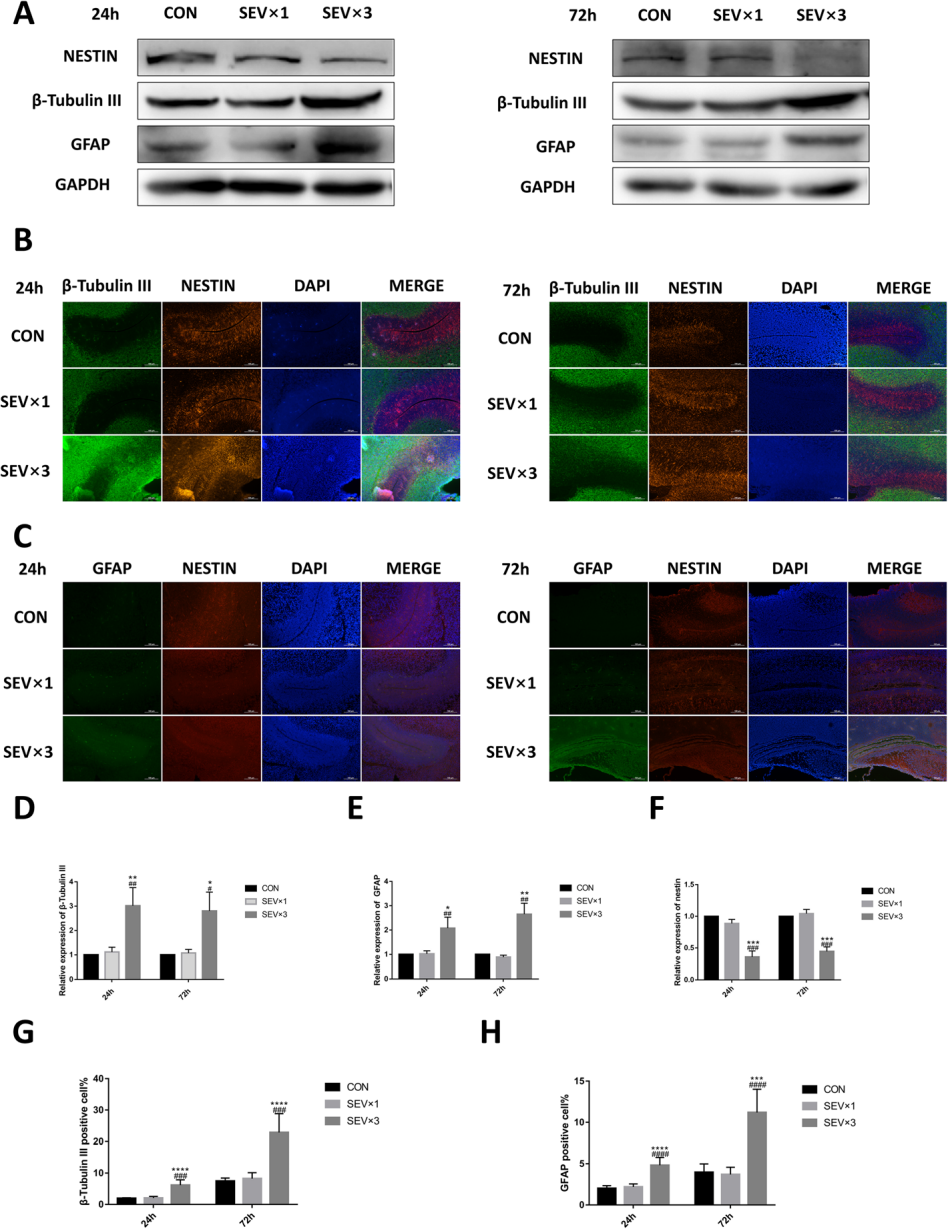
Repeated maternal exposure to 3% sevoflurane led to early NSC differentiation in fetal brains. **a** Western blotting images of β-tubulin III, GFAP, and nestin. **b** Immunofluorescence images of β-tubulin III (green) and nestin (red). Scale bar = 100 μm. **c** Immunofluorescence images of GFAP (green) and nestin (red). Scale bar = 100 μm. **d** Quantitative analysis of β-tubulin III. **e** Quantitative analysis of GFAP. **f** Quantitative analysis of nestin. **g** Quantification of β-tubulin III-positive cells. **h** Quantification of GFAP-positive cells. Values are means ± SD (*n* = 5/group). *p < 0.05, **p < 0.01, ***p < 0.001 compared with control (CON) group; # *p* < 0.05, ## *p* < 0.01, ### *p* < 0.001 compared with sevoflurane (SEV) × 1 group. One-way analysis of variance (ANOVA) followed by Tukey’s post hoc multiple comparison test was used for data analysis

**Fig. 3:**
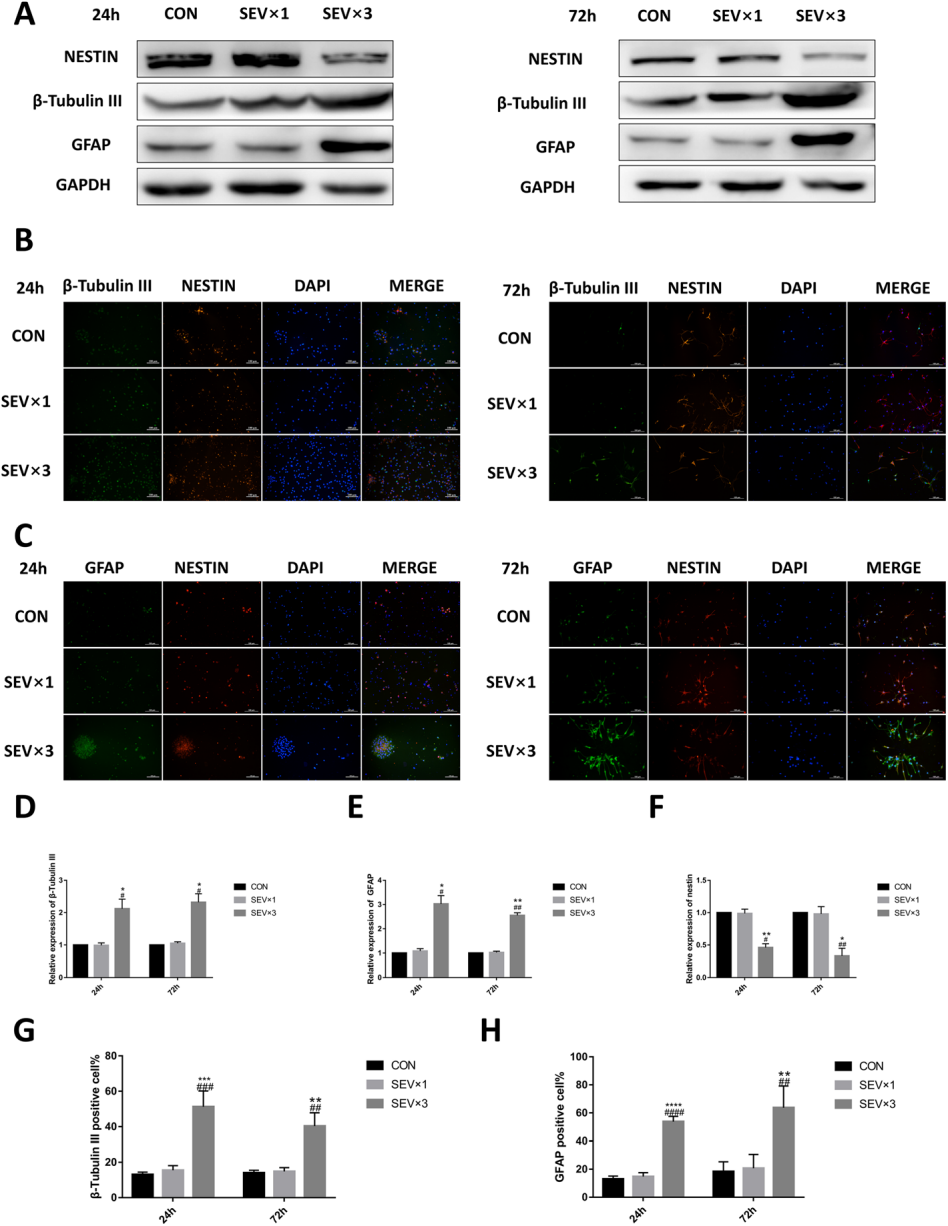
Repeated exposure to 4.1% sevoflurane led to early differentiation in primary cultured hippocampal NSCs. **a** Western blotting images of β-tubulin III, GFAP, and nestin. **b** Immunofluorescence images of β-tubulin III (green) and nestin (red). Scale bar = 100 μm. **c** Immunofluorescence images of GFAP (green) and nestin (red). Scale bar = 100 μm. **d** Quantitative analysis of β-tubulin III. **e** Quantitative analysis of GFAP. (F) Quantitative analysis of nestin. **g** Quantification of β-tubulin III-positive cells. **h** Quantification of GFAP-positive cells. Values are means ± SD (*n* = 3/group). *p < 0.05, **p < 0.01 compared with CON group; #*p* < 0.05, ##*p* < 0.01 compared with SEV × 1 group. One-way ANOVA followed by Tukey’s post hoc multiple comparison test was used for data analysis

To determine whether sevoflurane can affect NSC differentiation, the expression of NSC marker nestin, neuron marker β-tubulin III, and astrocyte marker GFAP was examined in fetal brains and primary cultured hippocampal NSCs using western blotting and immunofluorescence at 24 h and 72 h after single or repeated exposure to sevoflurane (Figs. 2 and 3). After repeated maternal exposure to 3% sevoflurane, the β-tubulin III (Fig. 2a, b, d, g) and GFAP levels (Fig. 2a, c, e, h) were increased and the nestin level (Fig. 2a–c, f) was decreased in fetal brain tissue. However, significant difference was not observed between control (CON) group and single 3% sevoflurane exposure (SEV × 1) group(Fig. 2a–h). Primary cultured NSCs exposed to 4.1% sevoflurane once or 3 times (SEV × 3) showed β-tubulin III (Fig. 3a, b, d, g), GFAP (Fig. 3a, c, e, h), and nestin (Fig. 3a, c, f) levels corresponded to in vitro results*.* The results showed that repeated exposure to sevoflurane led to early differentiation in hippocampal NSCs.

### Repeated exposure to sevoflurane led to long-term neuron reduction and astrocyte proliferation in hippocampus

**Fig. 4:**
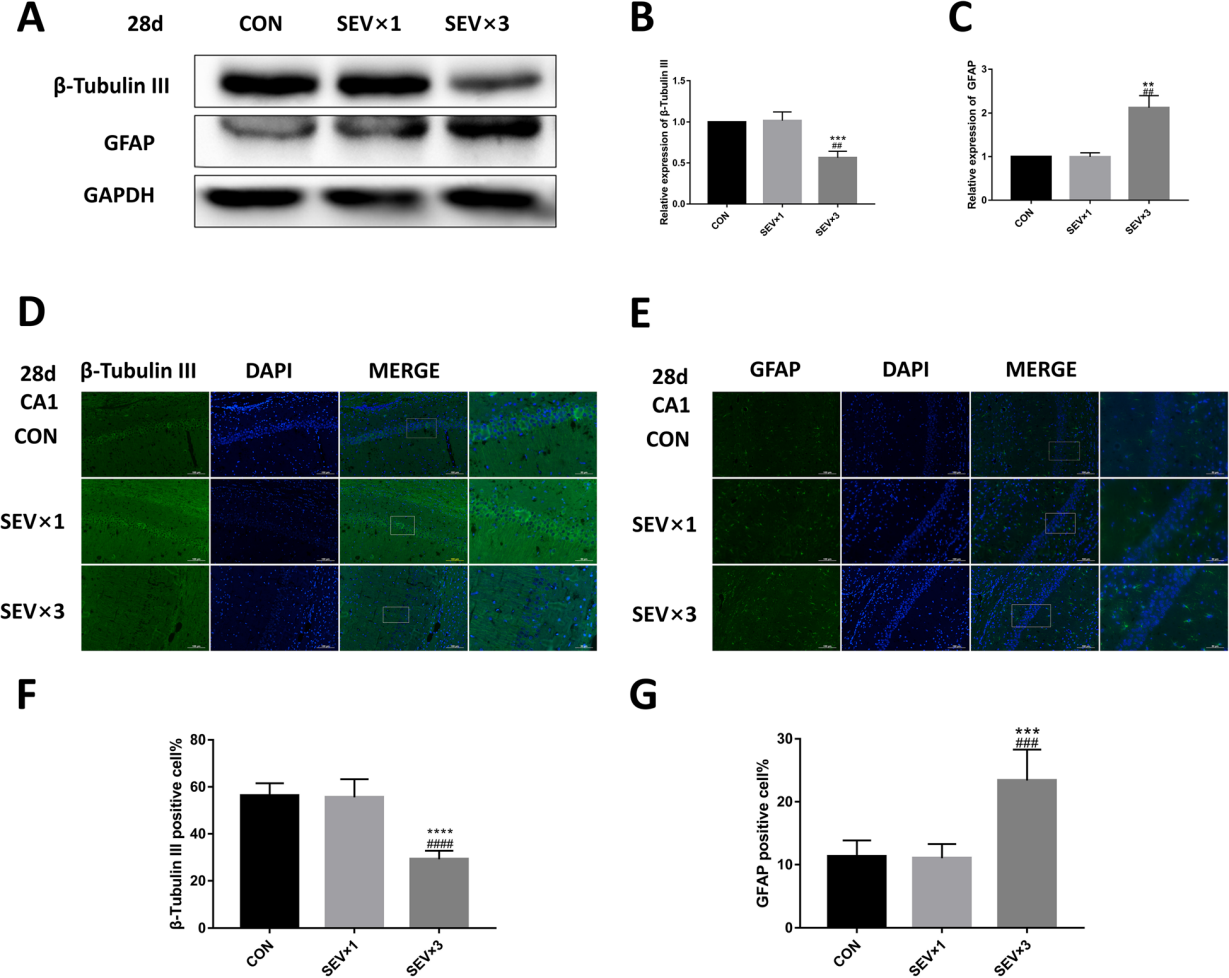
Effects of sevoflurane exposure on the expression of β-tubulin III and GFAP in fetal hippocampi on postnatal day 28. **a** Western blotting images of β-tubulin III and GFAP. **b** Quantitative analysis of β-tubulin III. **c** Quantitative analysis of GFAP. **d** Immunofluorescence images of β-tubulin III (green) in hippocampal CA1 region. Scale bar = 100 μm, 50 μm. **e** Immunofluorescence images of GFAP (green) in hippocampal CA1 region. Scale bar = 100 μm, 50 μm. **f** Quantification of β-tubulin III-positive cells. **g** Quantification of GFAP-positive cells. Values are means ± SD (*n* = 5). **p < 0.01, ***p < 0.001 compared with CON group; ##*p* < 0.01 compared with SEV × 1 group. One-way ANOVA followed by Tukey’s post hoc multiple comparison test was used for data analysis

**Fig. 5:**
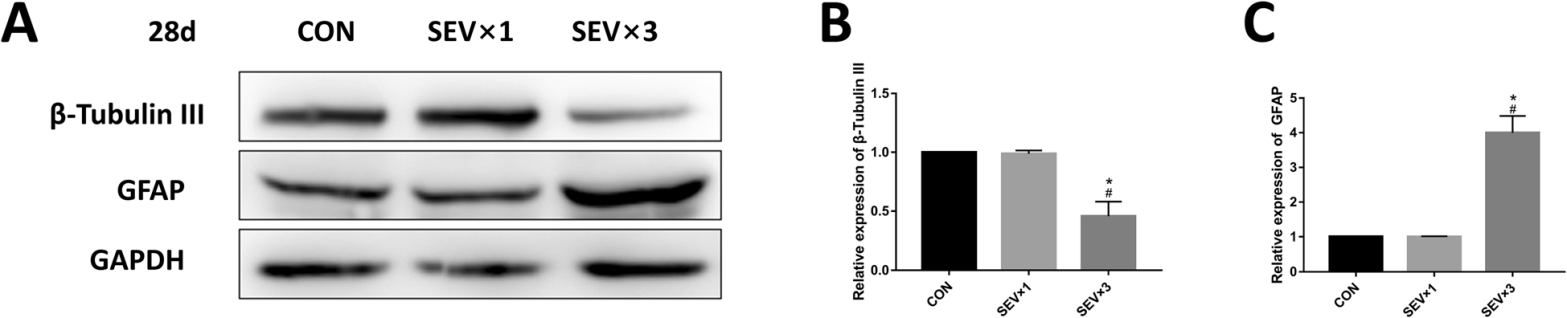
Effects of sevoflurane exposure on β-tubulin III and GFAP expression in cultured NSCs on postnatal day 28. **a** Western blotting images of β-tubulin III and GFAP. **b** Quantitative analysis of β-tubulin III. **c** Quantitative analysis of GFAP. Values are means ± SD (*n* = 3). *p < 0.05 compared with CON group; #*p* < 0.05 compared with SEV × 1 group. One-way ANOVA followed by Tukey’s post hoc multiple comparison test was used for data analysis

Our previous study showed that a single maternal 3% sevoflurane exposure does not cause long-term neurocognitive impairment in fetal rats, while repeated exposure of 3% sevoflurane can cause learning and memory impairment in the offspring [19]. The β-tubulin III and GFAP levels in rat brains were examined on postnatal day 28 (Fig. 4) as well as in the cultured NSCs (Fig. 5). After repeated exposure to sevoflurane, β-tubulin III protein was reduced in both postnatal fetal hippocampus (Fig. 4a, b) and cultured NSCs (Fig. 5a, b) compared with the CON group and SEV × 1 group. GFAP expression was upregulated in postnatal fetal hippocampus (Fig. 4a, c) and cultured NSCs (Fig. 5a, c). Immunofluorescence showed similar results regarding the quantity of neurons and astrocytes in CA1 hippocampal region (Fig. 4d–g). Significant difference was not observed between CON group and SEV × 1 group.

### Exposure to sevoflurane affected NSC differentiation by downregulating ATN1 expression

**Fig. 6:**
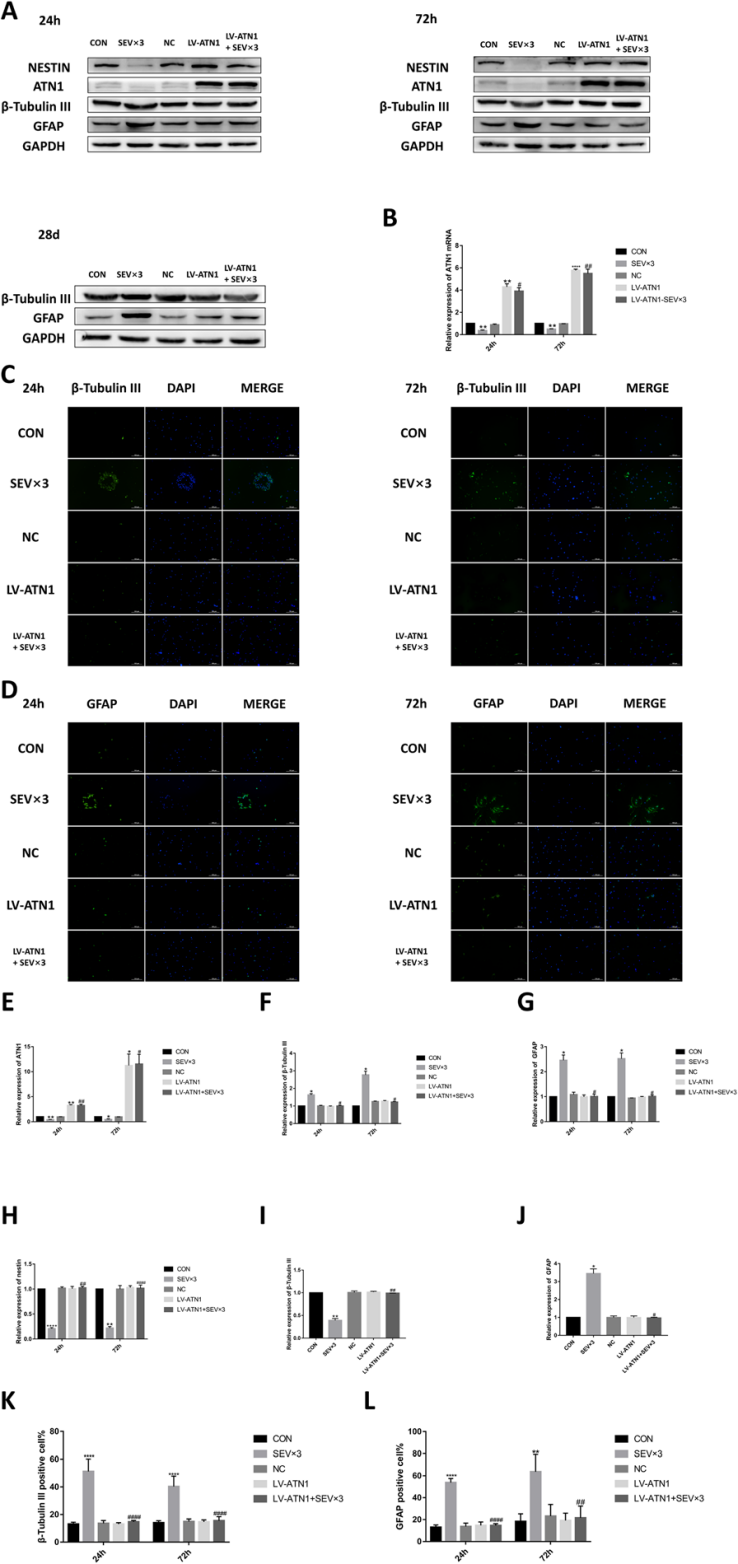
ATN1 overexpression alleviated sevoflurane-induced early NSC differentiation. **a** Western blotting images of β-tubulin III, GFAP, nestin, and atrophin 1(ATN1). **b** RT-qPCR analysis of ATN1 mRNA expression. **c** Immunofluorescence images of β-tubulin III (green). Scale bar = 100 μm. **d** Immunofluorescence images of GFAP (green). Scale bar = 100 μm. **e** Quantitative analysis of ATN1. **f** Quantitative analysis of β-tubulin III. **g** Quantitative analysis of GFAP. **h** Quantitative analysis of nestin. **i** Quantitative analysis of β-tubulin III on day 28. **j** Quantitative analysis of GFAP on day 28. **k** Quantification of β-tubulin III-positive cells. **l** Quantification of GFAP-positive cells

The expression of ATN1 was downregulated in both fetal brain tissues (Fig. S1A, B) and primary cultured NSCs (Fig. 6a, e) after repeated exposure to sevoflurane. After transfecting ATN1 overexpression lentivirus into NSCs and exposing to sevoflurane, in the 4.1% SEV × 3 + ATN1-overexpression lentivirus (LV-ATN1 + SEV × 3) group, the levels of β-tubulin III (Fig. 6a, c, f, k), GFAP (Fig. 6a, d, g, l), and nestin (Fig. 6a, h) were not significantly different than in the CON group at 24 h and 72 h. In the LV-ATN1 + SEV × 3 group, β-tubulin III (Fig. 6a, c, f, k) and GFAP (Fig. 6a, d, g, l) expressions were significantly reduced and nestin (Fig. 6a, h) expression was upregulated compared with the SEV × 3 group. ATN1 mRNA expression level was detected using reverse transcription quantitative polymerase chain reaction (RT-qPCR; Fig. 6b). β-tubulin III (Fig. 6a, i) and GFAP (Fig. 6a, j) protein levels were rescued in the LV-ATN1 + SEV × 3 group at day 28. Results confirmed that sevoflurane affected differentiation of hippocampal NSCs by downregulating ATN1 expression.

Values are means ± SD (*n* = 3). *p < 0.05, **p < 0.01, ****p < 0.0001 compared with CON group; #*p* < 0.05, ##*p* < 0.01, ####*p* < 0.0001 compared with SEV × 3 group. One-way ANOVA followed by Tukey’s post hoc multiple comparison test was used for data analysis.

### ATN1 is the direct target of miR-410-3p

**Fig. 7:**
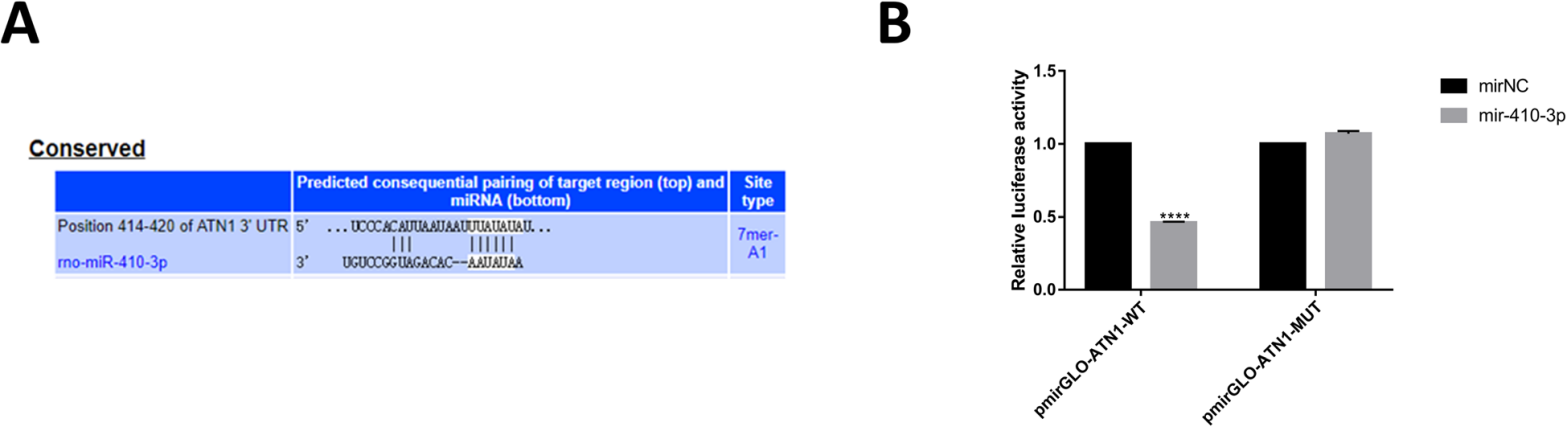
ATN1 is a target gene of miR-410-3p. **a** Target prediction program predicted a specific binding region between the ATN1 gene and miR-410-3p sequence. **b** Relative luciferase activity assay analysis. The experiment was performed three times. Paired Student’s *t* test was used for data analysis

Whether ATN1 is the direct target of miR-410-3p was investigated using TARGETSCAN database [20, 21]. The analysis showed ATN1 was the target site of miR-410-3p as described in Fig. 7a. The site was further verified using the dual-luciferase reporter assay (Fig. 7b).

### Exposure to sevoflurane affects NSC differentiation by upregulating miR-410-3p

**Fig. 8:**
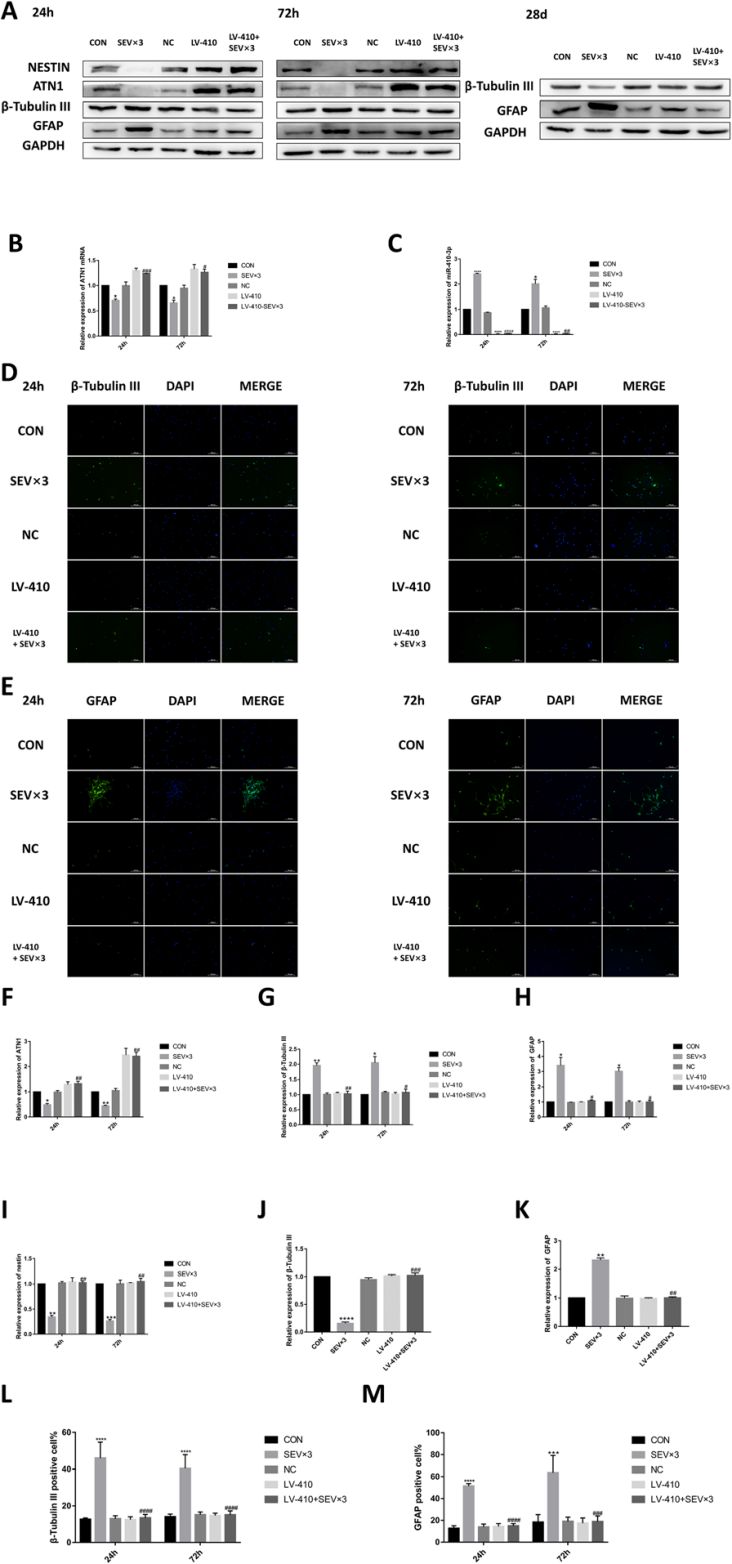
MiR-410-3p suppression alleviated sevoflurane-induced early NSC differentiation. **a** Western blotting images of β-tubulin III, GFAP, nestin, and ATN1. **b** RT-qPCR analysis of miR-410-3p expression. **c** RT-qPCR analysis of ATN1 expression. **d** Immunofluorescence images of β-tubulin III (green). Scale bar = 100 μm. **e** Immunofluorescence images of GFAP (green). Scale bar = 100 μm. **f** Quantitative analysis of ATN1. **g** Quantitative analysis of β-tubulin III. **h** Quantitative analysis of GFAP. **i** Quantitative analysis of nestin. **j** Quantitative analysis of β-tubulin III on day 28. **k** Quantitative analysis of GFAP on day 28. **l** Quantification of β-tubulin III-positive cells. **m** Quantification of GFAP-positive cells. Values are means ± SD (*n* = 3). *p < 0.05, **p < 0.01, ***p < 0.001, ****p < 0.0001 compared with CON group; #*p* < 0.05, ##*p* < 0.01, ###*p* < 0.001 compared with SEV × 3 group. One-way ANOVA followed by Tukey’s post hoc multiple comparison test was used for data analysis

The miR-410-3p level was upregulated in NSCs repeatedly exposed to sevoflurane. The levels of ATN1 (Fig. 8a, f), β-tubulin III (Fig. 8a, d, g, l), GFAP (Fig. 8a, e, h, m), and nestin (Fig. 8a, i) in NSCs treated with miR-410-3p suppression lentivirus and repeatedly exposed to sevoflurane were significantly different than levels in the SEV × 3 group at 24 h and 72 h. In the 4.1% SEV × 3 + miR-410-3p-suppression lentivirus (LV-410-SEV × 3) group, β-tubulin III expression (Fig. 8a, j) was upregulated and GFAP (Fig. 8a, k) expression was downregulated compared with the SEV × 3 group in cultured NSCs on day 28. RT-qPCR showed that miR-410-3p was suppressed by lentivirus (Fig. 8b). ATN1 mRNA level in LV-410 + SEV × 3 group was upregulated compared with the SEV × 3 group (Fig. 8c).

## Discussion

Numerous animal studies have shown that exposure to anesthesia drugs can lead to long-term neurocognitive impairment in developing brains [22, 23]. The FDA has published a warning that neurodevelopment of children’s brains may be affected in children under 3 years of age or pregnant women in the third trimester who undergo anesthesia more than once or for more than 3 h [24]. Mid-trimester is the most common time for non-obstetric surgery; however, the developing brain is in a vulnerable window [25, 26] and minor influences such as drugs and environment may cause severe neurocognitive impairment [27–29]. A safe surgery period does not equate to a safe anesthesia period. In the present study, the mechanism of NSC differentiation after exposure to sevoflurane during mid-trimester was investigated and advice provided for further clinical application.

Cell cycle timing dictates cell fate [30]. Premature NSC differentiation may have negative effects on developing brains. In several studies, premature differentiation of NSCs was shown to cause severe brain developmental disorders [31]. Research showed that aneuploidy causes NSCs to exit the cell cycle and differentiate prematurely, eventually resulting in the formation of microcephalic brains [32] In another study, *Nap1l1* knockdown caused less BrdU incorporation and greater expression of Tuj1^+^ cells (neuronal marker) in embryonic day (E) 13.5 fetal brains, resulting in developmental deficiencies [33]. In maternal diabetes model, the levels of circulating metabolite detoxifying enzyme glyoxalase 1 were decreased, which led to premature neurogenesis and adverse long-term influence on the developing brain of offspring [34].

Although some study results have shown certain anesthesia drugs may affect NSC differentiation, the findings are conflicting and controversial during different periods of brain development. For example, in a previous study, 4.1% sevoflurane for 6 h repressed neural differentiation by influencing self-renewal of mouse embryonic stem cells (mESCs) [35]. In another study, 7-day-old rats exposed to ketamine showed significantly reduced number of nestin/BrdU double-stained positive cells and GFAP/BrdU double-stained positive cells and increased number of β-tubulin III/BrdU double-stained positive cells [36]. Prolonged 2.4% isoflurane significantly suppressed neuronal fate while promoting glial fate in RcNcell CX human neural progenitor cells [37]. In the present study, 3% sevoflurane (1.5 minimum alveolar concentration, MAC) was chosen on pregnant rats, a commonly used concentration in clinical non-obstetric surgeries. 4.1% sevoflurane was commonly used in NSCs to explore the neurotoxicity of SEV in vitro in previous studies [35, 38]. There are other studies [39, 40] using 3% SEV in vivo and 4.1% SEV in vitro to investigate the mechanism of SEV. The results showed that during mid-trimester, multiple exposures to sevoflurane can cause premature differentiation of NSCs in developing brains of offspring. A single exposure to 3% sevoflurane did not significantly influence NSCs after 24 h and 72 h. After 3 times exposure to 3% sevoflurane, the expression of NSC marker nestin was downregulated; however, the expression of neuron marker β-tubulin III and astrocyte marker GFAP was upregulated in the early neurodevelopmental period. The results indicated that multiple exposures to anesthesia can reduce NSCs self-renewal capability and lead NSCs to early differentiation. Over an extended period, β-tubulin III was suppressed while GFAP remained overexpressed. The reduction of β-tubulin III indicates the number of neurons was reduced, which supports the same behavioral test results obtained in our previous research showing multiple exposures to sevoflurane can lead to neurocognitive impairment and learning disability in the offspring [19]. Assumedly, sevoflurane causes premature differentiation of NSCs which result in neuronal damage in postnatal off-spring. Consequently, due to the reduction of neurons, gliocytes, such as astrocytes, appear to undergo compensatory proliferation. However, the specific mechanism remains unknown.

Because sevoflurane is one of the most common anesthesia drugs used during pregnancy, the effects on neurodevelopment are crucial. In our previous research, results indicated that sevoflurane can affect proliferation of NSCs and leads to early NSC apoptosis in the offspring [6, 7]. β-tubulin III stained positive cells and GFAP stained positive cells in proportion to nestin stained positive cells of SEV × 3 group rates than CON group which confirms sevoflurane can cause early differentiation of NSCs. Although NSCs have proliferation ability, if they differentiate early to neurons, the proliferation process is disrupted and results in early reduction of neurons. The results from the present study indicate the number of neurons is reduced in the developing brain for an extended period of time after exposure to sevoflurane. Gliocytes tend to proliferate to compensate for the reduction of neurons but cannot reverse the effects on neurocognitive impairment.

The normal ATN1 expression is important for NSC maintenance [12]. Lower ATN1 expression indicates early differentiation of NSCs [12]. After exposure to sevoflurane, ATN1 expression was significantly reduced simultaneously with early NSC differentiation. To detect whether sevoflurane affects NSC differentiation by downregulating ATN1 expression, ATN1 overexpression lentivirus was transfected into primary rat NSCs. The results showed that ATN1 overexpression can reverse the effects of sevoflurane which confirms sevoflurane affected NSC differentiation by regulating ATN1 expression.

MiRNAs play an important role in brain development and miRNAs were affected after exposure to anesthesia drugs in several studies [41, 42]. To further explore the mechanism of NSC differentiation after exposure to sevoflurane, we predicted several possible binding sites using multiple target prediction programs [20, 21] and confirmed the sites through experiments. After exposure to sevoflurane, the miR-410-3p expression was significantly upregulated. Dual luciferase reporter assay showed that ATN1 is a direct target of miR-410-3p; miR-410-3p suppression can alleviate the effects of sevoflurane.

In the present study, the focus was on the mid-trimester of pregnancy which is the period when NSCs undergo significant proliferation and differentiation. Despite differences among studies, anesthesia drugs were shown in several reports to cause long-term neural impairment [43, 44]. In the present study, mid-trimester pregnant rats and primary cultured hippocampal NSCs extracted from the same period were used to investigate the influence of sevoflurane on hippocampal NSC differentiation.

The present study had several limitations. First, due to technical limitations, the in vivo mechanism was not investigated. Primary cultured NSCs were used to closely simulate the in vivo environment. Second, the density of cultured NSCs at day 28 was too low to perform immunofluorescence; however, western blot analysis was used to detect the expression of β-tubulin III and GFAP. Third, only a single inhalational anesthetic was used instead of a drug combination. Combined use of general anesthetics may cause diverse neurotoxicity outcomes, which may be the subject of our future investigations.

## Conclusions

In conclusion, the results from the present study demonstrated that repeated sevoflurane exposure during mid-trimester pregnancy caused early differentiation of NSCs by regulating miR-410-3p and ATN1 expression in the offspring. The results may provide several novel strategies for future clinical use.

## Supplementary information

**Additional file 1: Figure S1.** Effects of sevoflurane exposure on the expression of ATN1 in fetal hippocampi. (A) Western blotting images of ATN1 at 24 h. (B) Western blotting images of ATN1 at 72 h.

## Data Availability

All data generated or analyzed during this study are included in this published article and its supplementary information files.

## Abbreviations

NSC: Neural stem cell
G: Gestational day
GFAP: Glial fibrillary acidic protein
ATN1: Atrophin 1
MiRNAs: MicroRNAs
DRPLA: Dentatorubral-pallidoluysian atrophy
DMEM: Dulbecco’s modified Eagle’s medium
bFGF: Basic fibroblast growth factor
EGF: Epidermal growth factor
FBS: Fetal bovine serum
SEV: Sevoflurane
E: Embryonic
ANOVA: Analysis of variance
CON: Control group
SOX2: Sex-determining region Y box 2

## References

[1] Xu L, Shen J, Yu L, Sun J, Yan M. Autophagy is involved in sevoflurane-induced developmental neurotoxicity in the developing rat brain. Brain Res Bull. 2018;140:226–32. 10.1016/j.brainresbull.2018.05.014. 10.1016/j.brainresbull.2018.05.014 29803872

[2] Shenv FY, Song YC, Guo F, Xu ZD, Li Q, Zhang B, Liu ZQ. Cognitive Impairment and Endoplasmic Reticulum Stress Induced by Repeated Short-Term Sevoflurane Exposure in Early Life of Rats. Fron Psychiatry. 2018;9:332–2. 10.3389/fpsyt.2018.00332. 10.3389/fpsyt.2018.00332 PMC6083612 30116207

[3] Pearn ML, Schilling JM, Jian M, Egawa J, Wu C, Mandyam CD, Head BP. Inhibition of RhoA reduces propofol-mediated growth cone collapse, axonal transport impairment, loss of synaptic connectivity, and behavioural deficits. Br J Anaesth. 2018;120(4):745–60. 10.1016/j.bja.2017.12.033. 10.1016/j.bja.2017.12.033 PMC6200100 29576115

[4] Meredith RM, Dawitz J, Kramvis I. Sensitive time-windows for susceptibility in neurodevelopmental disorders. Trends Neurosci. 2012;35(6):335–44. 10.1016/j.tins.2012.03.005. 10.1016/j.tins.2012.03.005 22542246

[5] Silbereis JC, Pochareddy S, Zhu Y, Li M, Sestan N. The Cellular and Molecular Landscapes of the Developing Human Central Nervous System. Neuron. 2016;89(2):248–68. 10.1016/j.neuron.2015.12.008. 10.1016/j.neuron.2015.12.008 PMC4959909 26796689

[6] Wang Y, Yin S, Xue H, Yang Y, Zhang N, Zhao P. Mid-gestational sevoflurane exposure inhibits fetal neural stem cell proliferation and impairs postnatal learning and memory function in a dose-dependent manner. Dev Biol. 2018;435(2):185-97. 10.1016/j.ydbio.2018.01.022. 10.1016/j.ydbio.2018.01.022 29410165

[7] Li X, Wu Z, Zhang Y, Xu Y, Han G, Zhao P. Activation of Autophagy Contributes to Sevoflurane-Induced Neurotoxicity in Fetal Rats. Front Mol Neurosci. 2017;10:432. 10.3389/fnmol.2017.00432. 10.3389/fnmol.2017.00432 PMC5744904 29311820

[8] Wang Y, Yin SW, Zhang N, Zhao P. High-concentration sevoflurane exposure in mid-gestation induces apoptosis of neural stem cells in rat offspring. Neural Regen Res. 2018;13(9):1575–84. 10.4103/1673-5374.237121. 10.4103/1673-5374.237121 PMC6126114 30127118

[9] Schilling G, Wood JD, Duan K, Slunt HH, Gonzales V, Yamada M, Ross CA. Nuclear accumulation of truncated atrophin-1 fragments in a transgenic mouse model of DRPLA. Neuron. 1999);24(1):275-86. 10.1016/s0896-6273(00)80839-9 10677044

[10] Bidollari E, Rotundo G, Altieri F, Amicucci M, Wiquel D, Ferrari D, Rosati J. Generation of induced pluripotent stem cell line CSSi008-A (4698) from a patient affected by advanced stage of Dentato-Rubral-Pallidoluysian atrophy (DRPLA). Stem Cell Res. 2019;40:101551. 10.1016/j.scr.2019.101551. 10.1016/j.scr.2019.101551 31493762

[11] Napoletan F, Occhi S, Calamita P, Volpi V, Blanc E, Charroux B, Fanto M. Polyglutamine Atrophin provokes neurodegeneration in Drosophila by repressing fat. Embo j. 2011;30(5):945–58. 10.1038/emboj.2011.1. 10.1038/emboj.2011.1 PMC3049215 21278706

[12] Zhang F, Xu D, Yuan L, Sun Y, Xu Z. Epigenetic regulation of Atrophin1 by lysine-specific demethylase 1 is required for cortical progenitor maintenance. Nat Commun. 2014;5:5815. 10.1038/ncomms6815. 10.1038/ncomms6815 PMC4284801 25519973

[13] Rajman M, Schratt G. MicroRNAs in neural development: from master regulators to fine-tuners. Development. 2017;144(13):2310–22. 10.1242/dev.144337. 10.1242/dev.144337 28676566

[14] Shu P, Wu C, Liu W, Ruan X, Liu C, Hou L, Peng X. The spatiotemporal expression pattern of microRNAs in the developing mouse nervous system. J Biol Chem. 2019;294(10):3444–53. 10.1074/jbc.RA118.004390. 10.1074/jbc.RA118.004390 PMC6416447 30578296

[15] Shi Z, Zhou H, Lu L, Pan B, Wei Z, Liu J, Feng S. MicroRNA-29a regulates neural stem cell neuronal differentiation by targeting PTEN. J. Cell. Biochem. 2018;119(7):5813–20. 10.1002/jcb.26768. 10.1002/jcb.26768 29637609

[16] Morgado AL, Rodrigues CMP, Solá S. MicroRNA-145 Regulates Neural Stem Cell Differentiation Through the Sox2-Lin28/let-7 Signaling Pathway. Stem Cells. 2016;34(5):1386–95. 10.1002/stem.2309. 10.1002/stem.2309 26849971

[17] Shao CZ, Xia KP. Sevoflurane anesthesia represses neurogenesis of hippocampus neural stem cells via regulating microRNA-183-mediated NR4A2 in newborn rats. J Cell Physiol. 2019;234(4):3864–73. 10.1002/jcp.27158. 10.1002/jcp.27158 30191980

[18] Cao Se, Tian J, Chen S, Zhang X, Zhang Y. Role of miR-34c in ketamine-induced neurotoxicity in neonatal mice hippocampus. Cell Biol Int. 2015;39(2):164–8. 10.1002/cbin.10349. 10.1002/cbin.10349 25052764

[19] Wu Z, Li X, Zhang Y, Tong D, Wang L, Zhao P. Effects of Sevoflurane Exposure During Mid-Pregnancy on Learning and Memory in Offspring Rats: Beneficial Effects of Maternal Exercise. Front Cell Neurosci. 2018;12:122. 10.3389/fncel.2018.00122. 10.3389/fncel.2018.00122 PMC5943573 29773978

[20] Agarwal V, Bell GW, Nam JW, Bartel DP. Predicting effective microRNA target sites in mammalian mRNAs. eLife. 2015;4. 10.7554/eLife.05005. 10.7554/eLife.05005 PMC4532895 26267216

[21] Friedman RC, Farh KKH,, Burge CB, Bartel DP. Most mammalian mRNAs are conserved targets of microRNAs. Genome Res. 2009:19(1). 10.1101/gr.082701.108. 10.1101/gr.082701.108 PMC2612969 18955434

[22] Zhong Y, Chen J, Li L, Qin Y, Wei Y, Pan S, Xie Y. PKA-CREB-BDNF signaling pathway mediates propofol-induced long-term learning and memory impairment in hippocampus of rats. Brain Res. 2018;1691:64–74. 10.1016/j.brainres.2018.04.022. 10.1016/j.brainres.2018.04.022 29684336

[23] Han X, Liu C, Zhang K, Guo M, Shen Z, Liu Y, Li Y. Calpain and JNK pathways participate in isoflurane - induced nucleus translocation of apoptosis-inducing factor in the brain of neonatal rats. Toxicol Lett. 2018;285:60–73. 10.1016/j.toxlet.2017.12.022. 10.1016/j.toxlet.2017.12.022 29289695

[24] Olutoye OA, Baker BW, Belfort MA, Olutoye OO. Food and Drug Administration warning on anesthesia and brain development: implications for obstetric and fetal surgery. Am J Obstet Gynecol. 2018;218(1). 10.1016/j.ajog.2017.08.107. 10.1016/j.ajog.2017.08.107 28888583

[25] Pletikos M, Sousa AMM, Sedmak G, Meyer KA, Zhu Y, Cheng F, Sestan N. Temporal specification and bilaterality of human neocortical topographic gene expression. Neuron. 2014;81(2):321–32. 10.1016/j.neuron.2013.11.018. 10.1016/j.neuron.2013.11.018 PMC3931000 24373884

[26] Vasung L, Abaci Turk E, Ferradal SL, Sutin J, Stout JN, Ahtam B, Grant PE. Exploring early human brain development with structural and physiological neuroimaging. Neuroimage. 2019:187:226–54. 10.1016/j.neuroimage.2018.07.041. 10.1016/j.neuroimage.2018.07.041 PMC6537870 30041061

[27] Heroux NA, Horgan CJ, Rosen JB, Stanton ME. Cholinergic rescue of neurocognitive insult following third-trimester equivalent alcohol exposure in rats. Neurobiol Learn Mem. 2019:163:107030. 10.1016/j.nlm.2019.107030. 10.1016/j.nlm.2019.107030 PMC6689250 31185278

[28] Kanlikilicer P, Zhang D, Dragomir A, Akay YM, Akay M. Gene expression profiling of midbrain dopamine neurons upon gestational nicotine exposure. Med Biol Eng Compu. 2017;55(3):467–82. 10.1007/s11517-016-1531-8. 10.1007/s11517-016-1531-8 27255453

[29] Slotkin TA, Skavicus S, Card J, Levin ED, Seidler FJ. Diverse neurotoxicants target the differentiation of embryonic neural stem cells into neuronal and glial phenotypes. Toxicology. 2016:372:42-51. 10.1016/j.tox.2016.10.015. 10.1016/j.tox.2016.10.015 PMC5137195 27816694

[30] Dalton S. Linking the Cell Cycle to Cell Fate Decisions. Trends Cell Biol. 2015;25(10):592–600. 10.1016/j.tcb.2015.07.007. 10.1016/j.tcb.2015.07.007 PMC4584407 26410405

[31] Večeřa J, Procházková J, Šumberová V, Pánská V, Paculová H, Lánová MK, Pacherník J. Hypoxia/Hif1α prevents premature neuronal differentiation of neural stem cells through the activation of Hes1. Stem Cell Res. 2020;45:101770. 10.1016/j.scr.2020.101770. 10.1016/j.scr.2020.101770 32276221

[32] Gogendeau D, Siudeja K, Gambarotto D, Pennetier C, Bardin AJ, Basto R. Aneuploidy causes premature differentiation of neural and intestinal stem cells. Nat Commun. 2015;6:8894. 10.1038/ncomms9894. 10.1038/ncomms9894 PMC4660207 26573328

[33] Qiao H, Li Y, Feng C, Duo S, Ji F, Jiao J. Nap1l1 Controls Embryonic Neural Progenitor Cell Proliferation and Differentiation in the Developing Brain. Cell Rep. 2018;22(9):2279–93. 10.1016/j.celrep.2018.02.019.. 10.1016/j.celrep.2018.02.019 29490266

[34] Yang G, Cancino GI, Zahr SK, Guskjolen A, Voronova, A, Gallagher D, Miller FD. A Glo1-Methylglyoxal Pathway that Is Perturbed in Maternal Diabetes Regulates Embryonic and Adult Neural Stem Cell Pools in Murine Offspring. Cell Rep. 2016;17(4):1022–36. 10.1016/j.celrep.2016.09.067. 10.1016/j.celrep.2016.09.067 27760310

[35] Yi X, Cai Y, Zhang N, Wang Q, Li W. Sevoflurane inhibits embryonic stem cell self-renewal and subsequent neural differentiation by modulating the let-7a-Lin28 signaling pathway. Cell Tissue Res. 2016;365(2):319–30. 10.1007/s00441-016-2394-x. 10.1007/s00441-016-2394-x 27022747

[36] Huang H, Liu L, Li B, Zhao PP, Xu CM, Zhu YZ, Wu YQ. Ketamine Interferes with the Proliferation and Differentiation of Neural Stem Cells in the Subventricular Zone of Neonatal Rats. Cell Physiol Biochem. 2015;35(1):315–25. 10.1159/000369698. 10.1159/000369698 25591773

[37] Zhao X, Yang Z, Liang G, Wu Z, Peng Y, Joseph DJ, Wei H. Dual effects of isoflurane on proliferation, differentiation, and survival in human neuroprogenitor cells. Anesthesiology. 2013;118(3):537–49. 10.1097/ALN.0b013e3182833fae. 10.1097/ALN.0b013e3182833fae PMC3580019 23314167

[38] Liu S, Fang F, Song R, Gao X, Jiang M, Cang J. Sevoflurane affects neurogenesis through cell cycle arrest via inhibiting wnt/β-catenin signaling pathway in mouse neural stem cells. Life Sci. 2018;209:34–42. 10.1016/j.lfs.2018.07.054. 10.1016/j.lfs.2018.07.054 30071197

[39] Zhang Y, Lu P, Liang F, Liufu N, Dong Y, Zheng JC, Xie Z. Cyclophilin D Contributes to Anesthesia Neurotoxicity in the Developing Brain. Front Cell Dev Biol. 2019:7:396. 10.3389/fcell.2019.00396. 10.3389/fcell.2019.00396 PMC7026027 32117955

[40] Zhang J, Dong Y, Zhou C, Zhang Y, Xie Z. Anesthetic sevoflurane reduces levels of hippocalcin and postsynaptic density protein 95. Mol Neurobiol. 2015;51(3):853–63. 10.1007/s12035-014-8746-1. 10.1007/s12035-014-8746-1 24870966

[41] Jiang C, Logan S, Yan Y, Inagaki Y, Arzua T, Ma P, Bai X. Signaling network between the dysregulated expression of microRNAs and mRNAs in propofol-induced developmental neurotoxicity in mice. Sci Rep. 2018;8(1):14172. 10.1038/s41598-018-32474-3. 10.1038/s41598-018-32474-3 PMC6155049 30242182

[42] Bahmad HF, Darwish B, Dargham KB, Machmouchi R, Dargham BB, Osman M, Chamaa F. Role of MicroRNAs in Anesthesia-Induced Neurotoxicity in Animal Models and Neuronal Cultures: a Systematic Review. Neurotox Res. 2020;37(3):479–90. 10.1007/s12640-019-00135-6. 10.1007/s12640-019-00135-6 31707631

[43] Liu B, Ou G, Chen Y, Zhang J. Inhibition of protein tyrosine phosphatase 1B protects against sevoflurane-induced neurotoxicity mediated by ER stress in developing brain. Brain Res Bull. 2019;46:28–39. 10.1016/j.brainresbull.2018.12.006. 10.1016/j.brainresbull.2018.12.006 30553844

[44] Li GF, Li ZB, Zhuang SJ, Li GC. Inhibition of microRNA-34a protects against propofol anesthesia-induced neurotoxicity and cognitive dysfunction via the MAPK/ERK signaling pathway. Neurosci Lett. 2018;675:152–9. 10.1016/j.neulet.2018.03.052. 10.1016/j.neulet.2018.03.052 29578002

