# Supplementary figures and images for: Maternal sevoflurane exposure affects differentiation of hippocampal neural stem cells by regulating miR-410-3p and ATN1
Source: Stem Cell Res Ther. 2020 Sep 29;11:423. doi: 10.1186/s13287-020-01936-9 (PMC7523391; doi:10.1186/s13287-020-01936-9)

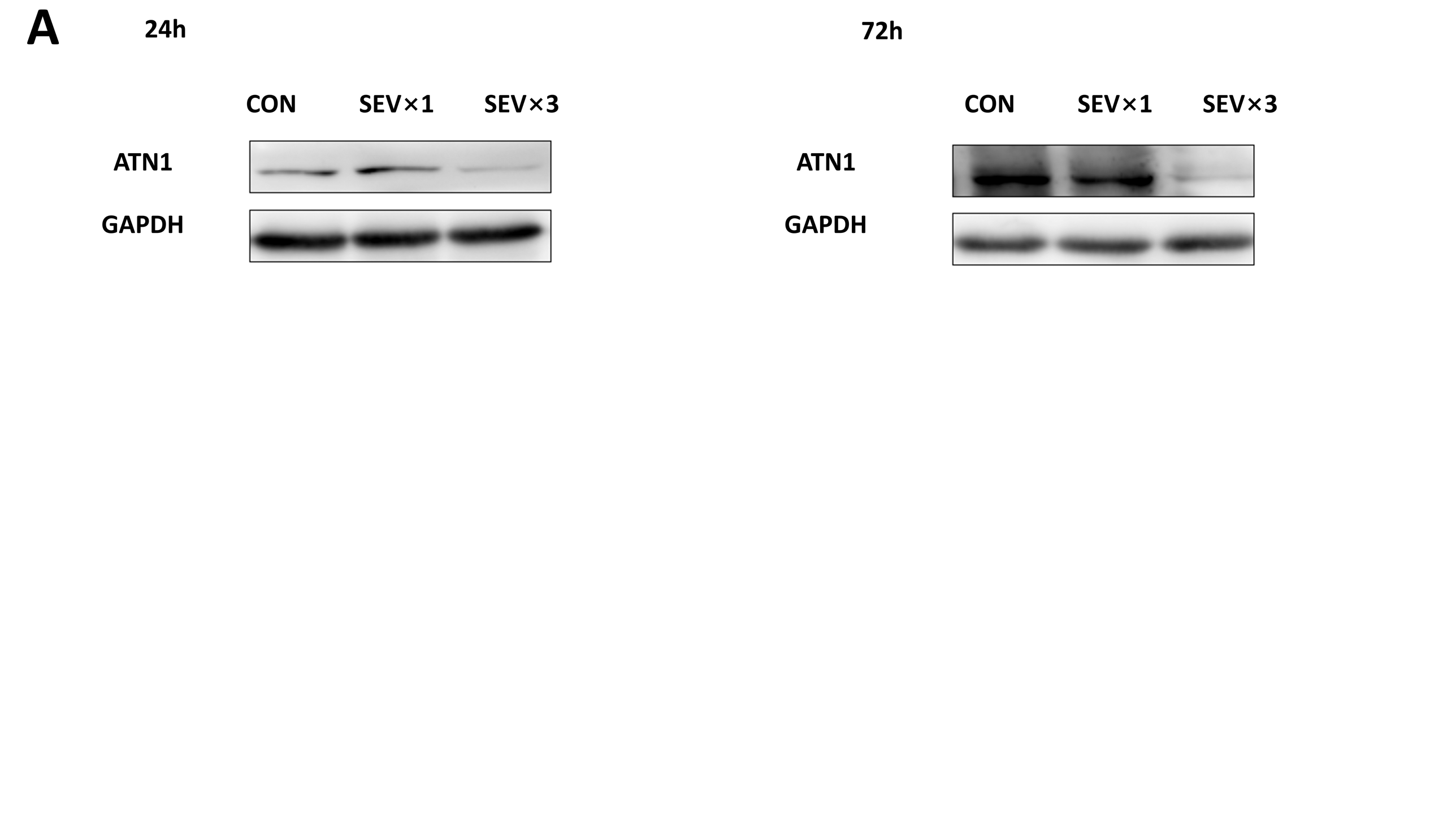

Supplement: Supplementary file 1 — Additional file 1: Figure S1. Effects of sevoflurane exposure on the expression of ATN1 in fetal hippocampi. (A) Western blotting images of ATN1 at 24 h. (B) Western blotting images of ATN1 at 72 h. [file 13287_2020_1936_MOESM1_ESM.png]
